# How Is the Cochlea Activated in Response to Soft Tissue Auditory Stimulation in the Occluded Ear?

**DOI:** 10.3390/audiolres11030031

**Published:** 2021-07-09

**Authors:** Miriam Geal-Dor, Haim Sohmer

**Affiliations:** 1 Speech & Hearing Center, Hebrew University-Hadassah Medical School, Jerusalem 91200, Israel; gmiriam@hadassah.org.il; 2 Department of Communication Disorders, Hadassah Academic College, Jerusalem 91200, Israel; 3 Department of Medical Neurobiology (Physiology), Hebrew University-Hadassah Medical School, P.O. Box 12272, Jerusalem 91120, Israel

**Keywords:** bone conduction, soft tissue conduction, occlusion effect, external canal, air conduction, vibrations, stethoscope

## Abstract

Soft tissue conduction is an additional mode of auditory stimulation which can be initiated either by applying an external vibrator to skin sites not overlying skull bone such as the neck (so it is not bone conduction) or by intrinsic body vibrations resulting, for example, from the heartbeat and vocalization. The soft tissue vibrations thereby induced are conducted by the soft tissues to all parts of the body, including the walls of the external auditory canal. In order for soft tissue conduction to elicit hearing, the soft tissue vibrations which are induced must penetrate into the cochlea in order to excite the inner ear hair cells and auditory nerve fibers. This final stage can be achieved either by an osseous bone conduction mechanism, or, more likely, by the occlusion effect: the vibrations of the walls of the occluded canal induce air pressures in the canal which drive the tympanic membrane and middle ear ossicles and activate the inner ear, acting by means of a more air conduction-like mechanism. In fact, when the clinician applies his stethoscope to the body surface of his patient in order to detect heart sounds or pulmonary air flow, he is detecting soft tissue vibrations.

## 1. Introduction

An auditory sensation can be initiated by several modes of auditory stimulation, each of which activates the hair cells and auditory nerve fibers of the inner ear.

### 1.1. Air Conduction

In most situations, hearing is elicited by alternating condensation rarefaction air pressures, initiated by the vibrations of the sound producing structure. The vibrations are conducted to the ear by air (hence called air conduction—AC). In the inner ear, the AC sound gives rise to an apparent mechanical wave progressing along the basilar membrane, which has been called the traveling wave.

### 1.2. Bone Conduction

(BC) is an additional mode of auditory stimulation induced when a clinical bone vibrator is applied to skin sites overlying skull bone such as at the mastoid or forehead, and initiates vibrations of skull bone. It is used mainly in the clinic in order to differentiate between a conductive hearing loss (CHL) (in which AC thresholds are elevated, but BC thresholds are in the normal range) and a sensorineural hearing loss (SNHL) (in which both AC and BC thresholds are elevated). The bone vibrations are conducted along skull bone to the outer, middle and inner ears (therefore, called bone conduction, a definition based on the medium through which the vibrations are conducted to the ear), where they give rise to the four generally accepted mechanisms of bone conduction acting simultaneously in parallel: the occlusion effect of the outer ear, inertia of the middle ear ossicles, inner ear fluid inertia and inner ear distortion (compression and expansion) [1]. These parallel BC mechanisms are thought to elicit hearing by eventually inducing a traveling wave along the basilar membrane, as in AC hearing [1]. BC is also used as an alternative form of hearing aid (bone anchored hearing aid—BAHA) in patients who cannot use a conventional AC hearing aid, with discharging ears and congenital malformations of the external ear [2,3].

### 1.3. Soft Tissue Conduction

Hearing can also be elicited by the relatively recently understood mode called soft tissue conduction, in which vibrations are initiated in the soft tissues of the body. The vibrations are induced either by an external vibrator (e.g., the clinical bone vibrator applied to skin sites not overlying skull bone, so that it is distinct from BC) or occur naturally, intrinsically in the body, e.g., by vibrations resulting from the heartbeat or blood flow [4] or vibrations of the vocal cord during self-vocalization, as described by von Bekesy [5]. These vibrations are conducted by the soft tissues to all parts of the body, including to the ear (therefore called soft tissue conduction—STC), and somehow excite it [6,7]. When the external auditory canal is occluded, the intrinsic vibrations become audible [4,7]. An example of STC can be demonstrated to the reader by occluding their external auditory canal with their finger in order to reduce possible external masking sounds, while gently stroking the stubble on the cheek or an ear ring. The auditory sensation perceived in response to the gentle stroking is due to STC (it is distinct from AC, since the external canal was occluded; and not BC, since bone vibrations were not induced).

In the past, all forms of hearing which were not directly initiated by AC had been traditionally grouped together under the general term “bone conduction”. However, given the present understanding of the nature of STC, it is now apparent that several auditory phenomena which had been originally referred to as being the result of BC, can now be shown to be elicited by STC: e.g., hearing one’s own voice [5,8], hearing of maternal sounds by the fetus in utero [9], and pulsatile tinnitus [10]. Therefore, while hearing by BC is mainly used in the clinic in order to differentiate between a CHL and a SNHL by assessing and comparing thresholds to AC and BC, the term “osseous BC” should be applied to those modes of hearing which are based on induction of actual vibrations of skull bone, and lead to the vibration of the outer, middle and inner ears [1].

### 1.4. Final Stage

However, the final stage of the hearing which is initiated by STC has yet to be demonstrated. In order for the soft tissue vibrations induced either by the external bone vibrator or intrinsically, e.g., by the heartbeat, to elicit an auditory sensation (hearing), the vibrations of the soft tissues must penetrate into the cochlea and excite the inner ear hair cells and the auditory nerve fibers. This final stage has been the source of several conflicting studies. Is the final stage of STC (inner ear excitation) achieved by inducing vibrations of actual skull bone, as in osseous BC, involving initiation of a traveling wave along the basilar membrane [11] (i.e., an osseous mechanism), or by an alternative non-osseous mechanism [6]? The answer to this question is important, since knowledge of the mechanism could contribute to improvements in the diagnosis of hearing loss assessed by the determination of the thresholds to AC and BC stimulation, and in the development of better forms of bone hearing aids.

## 2. Soft Tissue Conduction

The purpose of the present review is, therefore, to evaluate several alternative mechanisms which have been suggested to serve as the final stage of hearing initiated by STC, i.e., how the inner ear is excited in response to STC stimulation; and how the vibrations of the soft tissues, which result from the delivery of a vibratory stimulus to sites on the skin (not overlying skull bone) or initiated intrinsically in the body (for example, by contractions of the heart), reach and excite the inner ear.

### 2.1. Acoustic Impedance

Can the low magnitude soft tissue vibrations induced by threshold intensity STC stimulation give rise to skull bone vibrations? In other words: can, for example, the gentle stroking of the stubble on the cheek or the intrinsic sounds coming from the heartbeat or blood flow [4] eventually induce vibrations of the more rigid dense skull bone? This question can be expressed in physical terms by considering the acoustic impedances (defined as the product of the density of the medium and the velocity of sound in that medium) of the conducting media involved. The acoustic impedance of bone is 7.8 × 10^6^ kg/m^2^ s; of typical soft tissues is 1.6 × 10^6^ kg/m^2^ s; of water is 1.48 × 10^6^ kg/m^2^ s; and of air is 0.0004 × 10^6^ kg/m^2^ s [12,13,14]. When the acoustic impedances of two contiguous media are similar, the vibrations in one media are efficiently conducted to the other. However, when they differ (described as an impedance mismatch), the vibrations are attenuated at the interface. For example, at an air–water interface, an AC sound would be attenuated by about 30 dB, and not penetrate into the water [14]. In this case, the middle ear serves as an impedance matching device [12]. Given the differences in acoustic impedance between soft tissue and bone, the vibrations of the soft tissues would theoretically be attenuated by about 70% (equivalent to about 7 dB) at the soft tissue–bone interface [13]. This attenuation can be overcome by elevating the magnitude of the vibrations of the soft tissues, for example, by increasing the intensity of the stimulus acting on the soft tissues.

This theoretical degree of attenuation has been confirmed in studies conducted in the course of the development of BAHAs: the thresholds of BAHA patients to the more conventional application of the bone vibrator to the skin over the bone were compared to the thresholds of the same patients to the delivery of the vibratory stimuli directly to the bone BAHA titanium implant after it had been integrated into the bone. The two stimulation sites were 2 cm apart. The thresholds to the stimulus delivered directly to the implant were about 10 dB lower (better) than those delivered at the nearby skin [2]. In a complementary experiment, the magnitudes of the acceleration levels at hearing threshold in response to the delivery of the vibratory stimulus to the skin were measured on the titanium implant, and were compared to those measured on the nearby intact skin. The acceleration levels were about 20 dB lower (better) at threshold when measured directly on the implant [3]. In other words, the intensity of the vibratory stimuli delivered to the skin would have had to be about 10 dB greater than those delivered directly to the bone (i.e., the implant) in order to enable the stimulus to the skin overlying the bone to reach threshold and (apparently) induce vibrations of the underlying bone.

### 2.2. Is Bone Conduction the Final Stage?

A recent study [11] made use of the titanium implant integrated in the mastoid bone of five BAHA patients in order to measure the magnitude of the skull vibrations on the implant in response to several intensities of vibratory stimulation delivered to a soft tissue site (neck), which represents STC. In the same patients, behavioral thresholds were also assessed to the same stimuli applied at the neck site. The amplitudes of the bone vibrations were found to be linearly related to the STC (neck) stimulus intensities. However, the authors were unable to detect vibrations at actual behavioral threshold due to the inherent background noise accompanying body activity in live human patients (e.g., respiration, circulation, movements). The lowest intensity at which vibrations could be detected in response to stimulation at the neck in all five of the participants was 50 dB HL, and vibrations could be detected in three of the five participants at 30 dB HL. The vibration magnitudes measured on the implant in response to higher intensity stimuli were therefore linearly extrapolated by the authors down to the intensity which had been the behavioral threshold of the same participants, in order to obtain an estimate of the magnitude of the vibrations at threshold. The authors concluded from the linearity that STC (neck) thresholds were directly elicited by bone vibrations, i.e., by an osseous mechanism. However, this cannot be taken as evidence that the final stage of STC is osseous, since, though linearly related to the intensity of the STC stimulation at the neck, at some low level of the vibrations of the soft tissue, the magnitude of these vibrations would be below threshold; a non-osseous mechanism cannot be excluded; and the linear extrapolation may not provide the magnitude of the vibrations at actual threshold. Furthermore, as described above, the vibratory stimuli delivered to the skin overlying the mastoid bone would have to be about 10 dB greater in intensity in order to reach the threshold by an osseous mechanism. Therefore, all the more so, the vibrations within the soft tissues which had been elicited in response to threshold-level stimuli delivered to the neck STC site would surely not be able to induce vibrations of the mastoid bone, and a non-osseous mechanism is likely involved. It has been suggested that this is due to the soft tissues acting as a “shunt” for the vibratory stimuli [3], i.e., part of the vibratory energy was “dispersed” in the soft tissues, and not transmitted to the bone (as a result of the impedance mismatch). Also, since the attenuation of the STC-induced soft tissue vibrations at the soft tissue–bone interface is of the order of only 7 dB, the transition from a non-osseous mechanism which is effective at actual threshold to an osseous mechanism at some supra-threshold intensity may be undetectable when using 5 dB intensity steps.

Furthermore, in several examples of STC, additional evidence can be presented which suggests that the final stage of hearing may not involve an osseous mechanism. For instance, the fetus in utero, after about 20 weeks gestation, responds to maternal sounds. While these signs of fetal hearing have been ascribed to BC [9,15], it is now clear that the maternal sounds reach the fetus through the maternal and fetal soft tissues by STC [16]. However, the presence of amniotic fluid filling the fetal middle ear cavity [17], converts the impedance of the oval and round windows more similar to each other. Therefore, fetal hearing probably does not involve the BC mechanisms of inner ear fluid inertia and inner ear distortion, which are based on differences in the impedances between the two windows. In other words, the major osseous BC mechanisms effective in the adult ear [1], are greatly reduced in the fetal ear [18]. Furthermore, fetal skull bone is not fully developed, and there are membranous sutures between the component skull bones; hence, it likely would not be able to conduct vibrations directly along skull bone by bone conduction to the ear [19].

### 2.3. Occlusion Effect

**Figure 1:**
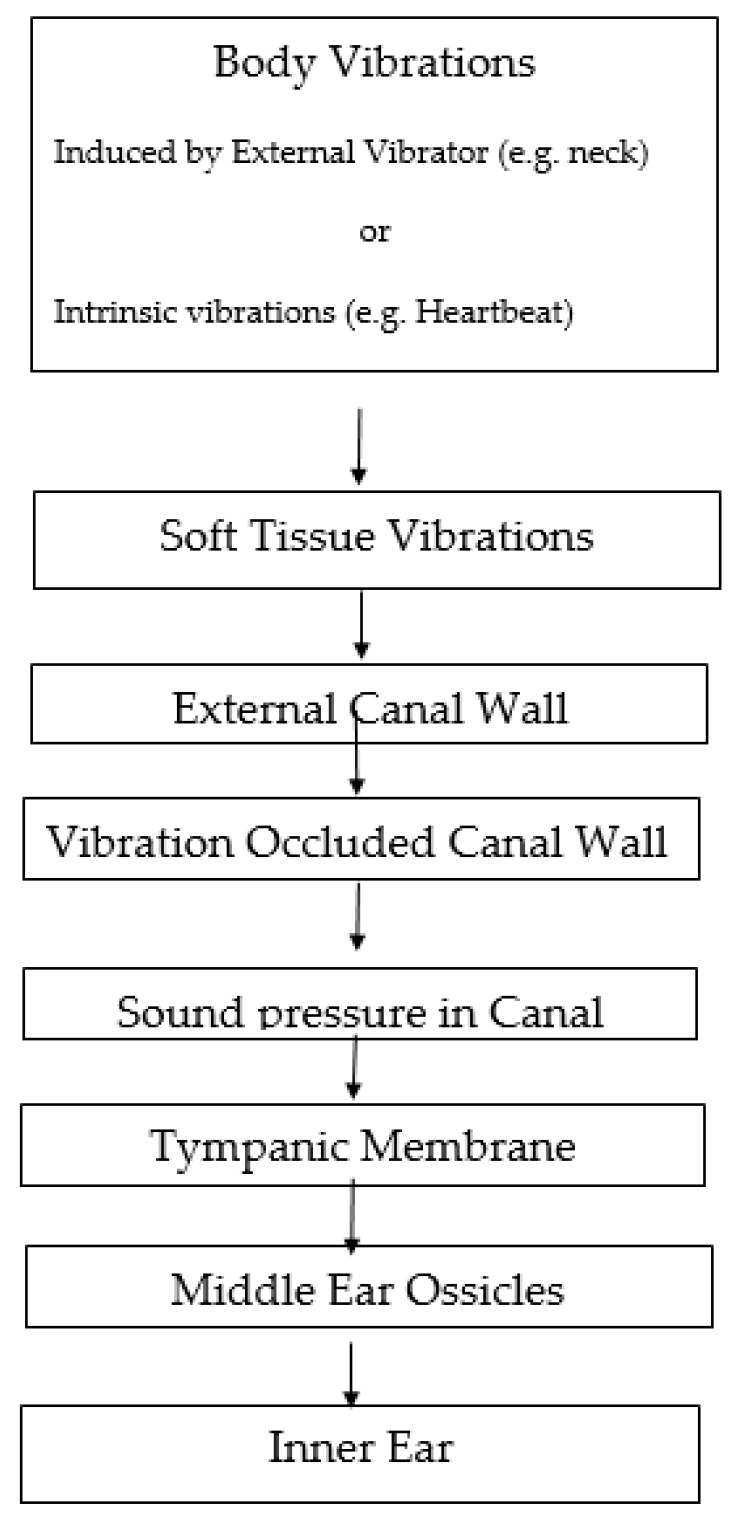
Schematic diagram showing the suggested mechanism of the final stage of hearing in response to soft tissue conduction: the vibrations of the soft tissues (initiated either by an external vibrator, for example, at the neck or by intrinsic body vibrations, e.g., heartbeat) elicit the occlusion effect, which produces sound pressure in the occluded external canal, and drive the tympanic membrane and the ossicular chain. Therefore, the inner ear is excited by a pathway similar to that in response to AC stimulation.

In addition, in studies designed to elucidate the mechanisms of STC hearing [7,20,21,22], the external auditory canal of the participants was usually occluded with an ear plug in order to exclude the possibility that the participant would respond to the AC sounds accompanying the STC stimulus delivered by a bone vibrator, and in order to reduce external masking sounds. However, in the presence of the occluding ear plugs, the occlusion effect (OE) would likely be elicited. It has been shown that the OE results from vibrations of the walls of the external auditory canal [23,24] which are induced by the vibrations of the soft tissues initiated by the external bone vibrator, or by the intrinsic body sounds resulting, for example, from the heartbeat. In fact, when the clinician uses a stethoscope to detect these intrinsic body sounds (e.g., heartbeat, pulmonary air flow) in their physical examination of a patient, they are making use of the intrinsic vibrations, and this serves as a clear and obvious confirmation of the existence of soft tissue vibrations and soft tissue conduction [7]. Since soft tissue (skin) provides the immediate lining of the cavity of the canal including both the cartilaginous and the bony parts of the canal, it is likely that the OE is the result of the vibrations of the more compliant soft tissue-cartilaginous walls of the canal [7]. These vibrations produce air pressures in the occluded cavity which drive the tympanic membrane and the middle ear ossicles, and excite the inner ear by a mechanism similar to that in response to AC stimulation [7]. Furthermore, the hearing of self-vocalizations [5,8] and of one’s own heartbeat and blood flow [4] when the external canal is occluded is also a result of the OE, in which the vibrations of the vocal cords during vocalization or the vibrations of the heart and blood flow are conducted by the soft tissues to the walls of the external auditory canal, leading to its vibration. It has also been shown that the OE elicited in response to the low frequency vibrations induced by the heartbeat and resulting blood flow reaches a magnitude of 40 to 50 dB [4], i.e., the sound pressure in the occluded external canal is 100 times greater than that in the open canal; and this would require relatively large excursions of an extensive area of the canal wall, probably the more compliant soft tissue-cartilaginous wall. The air pressures induced in the occluded canal drive the tympanic membrane and the middle ear ossicles, in a pathway similar to that in AC hearing [7] (see Figure 1). In addition, in the studies conducted on BAHA participants [11,25], the external auditory canal in the tested ear was occluded with an ear plug. Therefore, the OE was likely elicited, enhancing the sensitivity (reducing the threshold) of the BAHA participant. Thus, the STC vibrations induced by the bone vibrator at the neck STC site were conducted by means of STC to the external canal walls, causing their vibration. In the presence of the occluding ear plug, the sound pressure in the occluded ear canal would be elevated, driving the tympanic membrane, middle ear ossicles, exciting the inner ear by a mechanism based on a sequence of events similar to an AC pathway, i.e., leading to a traveling wave. Thus, while the OE is considered one of the component mechanisms of BC [1], the OE is likely the result of the vibrations of the soft tissue-cartilaginous part of the canal wall [7], and not of the bony part. This may also be the mechanism leading to hearing in response to the delivery of vibratory stimuli to fluid applied to the external canal (which is also a form of STC), which was effective mainly to the lower frequencies [26].

## 3. Conclusions

In conclusion, the OE enables the vibrations of the soft tissues to penetrate into the cochlea by means of the sound pressures in the occluded canal, and likely contributes a major component to the final stage of hearing in response to threshold intensity STC auditory stimulation.

## Data Availability

Not applicable.
